# Risk scores in cardiac resynchronization therapy–A review of the literature

**DOI:** 10.3389/fcvm.2022.1048673

**Published:** 2023-01-17

**Authors:** András Mihály Boros, Péter Perge, Béla Merkely, Gábor Széplaki

**Affiliations:** ^1^Heart and Vascular Center, Semmelweis University, Budapest, Hungary; ^2^Heart and Vascular Centre, Mater Private Hospital, Dublin, Ireland; ^3^Royal College of Surgeons in Ireland, Dublin, Ireland

**Keywords:** CRT, cardiac resynchronization therapy, prediction model, risk scores, mortality, response

## Abstract

Cardiac resynchronization therapy (CRT) for selected heart failure (HF) patients improves symptoms and reduces morbidity and mortality; however, the prognosis of HF is still poor. There is an emerging need for tools that might help in optimal patient selection and provide prognostic information for patients and their families. Several risk scores have been created in recent years; although, no literature review is available that would list the possible scores for the clinicians. We identified forty-eight risk scores in CRT and provided the calculation methods and formulas in a ready-to-use format. The reviewed score systems can predict the prognosis of CRT patients; some of them have even provided an online calculation tool. Significant heterogeneity is present between the various risk scores in terms of the variables incorporated and some variables are not yet used in daily clinical practice. The lack of cross-validation of the risk scores limits their routine use and objective selection. As the number of prognostic markers of CRT is overwhelming, further studies might be required to analyze and cross-validate the data.

## Introduction

According to the most recent guidelines, cardiac resynchronization therapy (CRT) is recommended for symptomatic heart failure patients in sinus rhythm with a QRS duration ≥150 ms and left bundle branch block (LBBB) QRS morphology and with left ventricular ejection fraction (LVEF) ≤35% despite optimal medical therapy to improve symptoms and reduce morbidity and mortality (1, 2). However, mortality is still high; and approximately one-third of the patients do not respond to CRT as adequately as expected, in whom no quality of live improvement or reverse remodeling of the left ventricle is seen (3).

Consequently, there is a great need for tools that might help in optimal patient selection and provide prognostic information for the patients and their families. Ever since the first implementation of CRT, several clinical factors and biomarkers have been tested in prediction models to identify those patients who might benefit the most from the therapy (4, 5). Prediction models are useful to reveal which parameters are statistically significant in the outcome prediction by giving the hazard and odds ratios, but they are not interpretable at the level of the individual patient in the clinical practice. Therefore, risk scores have been developed that constitute predominantly categorized variables with attributed points. The sum of the points reveals the exact risk of the individual; so that, patients can be easily and quickly grouped into risk categories with meaningful information.

Several risk scores have been created in CRT in recent years; however, no literature review is available that would list the possible scores for the clinicians.

Therefore, we aimed to systematically review the risk scores in CRT and provide the calculation methods and formulas in a ready-to-use format.

## Materials and methods

**FIGURE 1:**
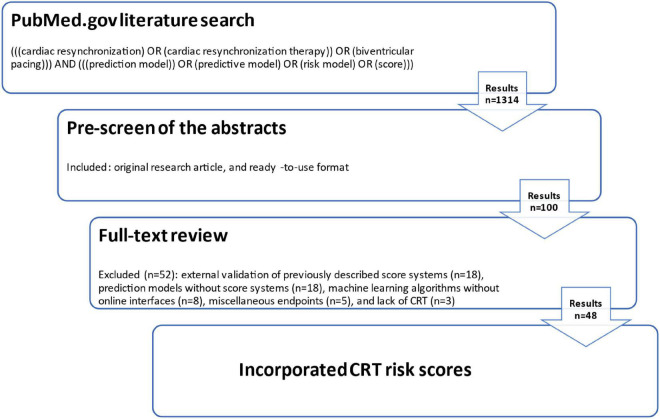
Flowchart of the review process.

The literature search was performed in November 2021 and then updated in September 2022 by using the search engine PubMed.gov^1^ with the input of the following equation: (((cardiac resynchronization) OR (cardiac resynchronization therapy)) OR (biventricular pacing))) AND (((prediction model)) OR (predictive model) OR (risk model) OR (score))). The flowchart of the review process is presented by Figure 1.

Since we applied no language or publication date restrictions, the result was 1,314 possible papers. Two investigators (AB and PP) independently pre-screened the abstracts of these manuscripts by considering further inclusion criteria: original research article, and ready-to-use format. This resulted in a sum of 100 records that were further assessed by full-text review. A total of 52 papers were excluded based on the following reasons: external validation of previously described score systems (*n* = 18), prediction models without score systems (*n* = 18), machine learning algorithms without online interfaces (*n* = 8), miscellaneous endpoints (*n* = 5), and lack of CRT (*n* = 3). Consequently, forty-eight CRT risk scores were incorporated into the present review.

## Results

**FIGURE 2:**
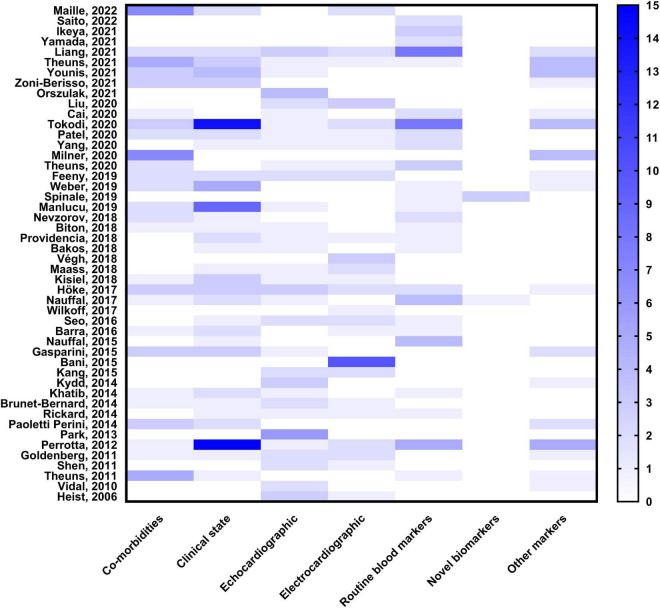
Heat map of the predictors used in the risk scores of cardiac resynchronization therapy.

To date, we identified 48 ready-to-use risk scores in heart failure patients with CRT Table 1. Summarizes the details of the models with the interpretation of the results and presents the formulas or the calculation methods of the scores Figure 2. Overviews the risk scores and helps in the selection of the appropriate risk score by considering the available data about the patient.

**TABLE 1:** Risk scores in cardiac resynchronization therapy.

References	Study pop.	Num. of pat.	Primary endpoint	Duration (months)	Score	Score details	Results
Heist et al. (6)	CRT	39	Δdp/dt > 25% of mitral regurgitation jet	acute	Response score	4 parameters, 0–4 points	There was a significant association between response score (0 to 4 points) and acute hemodynamic response to CRT (*p* < 0.0001).
Response score’s calculation: LV/right ventricular distance ≥ 10 cm, LV lead electrical delay ≥ 50%, baseline maximum ΔdP/dt ≤ 600 mm Hg/s, maximum time difference ≥ 100 ms. One point was attributed to each predictor.							
Vidal et al. (37)	CRT	147	Alive, no HTX + Δ6-min ≥ 10%	12		3 variables, score: 0–3	Patients with higher scores showed a significantly higher likelihood of non-response to CRT (*x*^2^ = 12 891, *p* = 0.005). Rates of response ranged from 80% for patients who scored 0 to 25% in patients with a score of 3.
Calculation: LVEDV ≥ 200 mL, mitral regurgitant orifice area ≥ 16 mm^2^, and score in the Minnesota questionnaire ≥ 41. One point was attributed to each predictor.							
Goldenberg et al. (9)	CRT-D, ICD	1,761	All-cause death ± HF hospitalization	12	MADIT-CRT score	7 parameters, risk score 0–14 points	Multivariate analysis showed a 13% (*p* < 0.001) increase in the clinical benefit of CRT-D per 1-point increment in the response score.
MADIT-CRT score’s calculation: female sex (2 points), non-ischemic origin (2 points), LBBB (2 points), QRS ≥ 150 ms (2 points), prior hospitalization for HF (1 point), LVEDV ≥ 125 mL/m^2^ (2 points), and LA volume ≥ 40 mL/m^2^ (3 points).							
Shen et al. (38)	CRT	100	ΔLVESV ≥ 15% reduction after 6-month	24		3 parameters, risk score 0–4 points	Cardiac resynchronization therapy responders in patients with response score > 2 and ≤ 2 were 36/38 (95%) and 7/62 (11%, *p* < 0.001), respectively.
Calculation: 1 point for RV pacing-induced LBBB, 1 point for wall motion score index ≤ 1.59, and 2 points for time difference between LV ejection measured by tissue Doppler and pulsed wave Doppler > 50 ms.							
Theuns et al. (8)	CRT-D	463	All-cause death	36	Charlson comorbidity index (CCI)	17 comorbid conditions, online calculator https://www.mdcalc.com/charlson-comorbidity-index-cci	CCI score ≥ 5 was a predictor of mortality (hazard ratio 3.69, 95% CI 2.06–6.60; *p* < 0.001) independent of indication for ICD therapy, and from ICD interventions during the clinical course.
CCI’s calculation: myocardial infarction, cerebrovascular disease, chronic obstructive pulmonary disease, diabetes, peripheral vascular disease, renal failure, and any malignancy excluding metastatic tumors. The comorbidity index was calculated by assigning a weight of 2 to renal failure and any malignancy, and a weight of 1 to the other comorbid conditions. The comorbidity score for each patient is the arithmetic sum of the value assigned to each identified comorbid condition. To account for the effects of increasing age, the comorbidity score was adjusted by adding one point to the score for each decade of life over the age of 50 at the time of implantation.							
Perrotta et al. (14)	CRT	342	All-cause death ± HTX ±	24	Seattle Heart Failure Model (SHFM)	25 parameters, online calculator https://depts.washington.edu/shfm/?width=1360&height=768	The SHFM was a good fit of death from any cause/cardiac transplantation, without significant differences between observed and SHFM-predicted survival.
SHFM’s calculation: age (years); weight (kg); gender (male/female); ischemic etiology (yes/no); NYHA (1–4); LVEF (%); systolic blood pressure (mm Hg); aldosterone blocker use (yes/no); statin use (yes/no); allopurinol use (yes/no); ACEI use (yes/no); ARB use (yes/no); diuretic dose/kg: furosemide, bumetanide, torsemide, metolazone, hydrochlorothiazide, chlorothiazide; hemoglobin (g/dL); lymphocyte count (%); uric acid (mg/dL); sodium (meq/L); total cholesterol (mg/dL); intravenous diuretics (yes/no); pressors (number); intra-aortic balloon pump, ventilator, ultrafiltration (yes/no); ICD, CRT-P, CRT-D (yes/no); wide QRS (yes/no), LBBB (yes/no).							
Park et al. (17)	CRT	334	ΔLVESV ≥ 15% reduction after 12-month	12	EchoCG score	6 parameters, including strain analysis, risk score of 0–37 points	Total score of > 17 (95% CI: 13–17) showed optimal sensitivity (84%) and specificity (79%) for response.
EchoCG score’s calculation: LA area < 26 cm^2^ = 1 point, intermediate for RV end-diastolic area index < 10.0 cm^2^/m^2^ = 2 points, RA area < 20 cm^2^ = 2 points, LV end-diastolic dimension index < 3.1 cm/m^2^ = 6 points, LVGLS < –7.0% = 6 points, RVFAC ≥ 35% = 20 points.							
Kydd et al. (18)	CRT	294	ΔLVESV ≥ 15% reduction after 6-month	24		3 parameters, including strain analysis. The *p*-score ranged from -1.1 to 9.4	A *p*-score > 3.28 offered high specificity (specificity 86%, sensitivity 70%) to predict response.
Calculation: [0.022 × IVMD (ms)] + [0.034 × RSD (%)] – [0.13 × LVGLS (%)] – [2.3 if suboptimal LV lead, 0 if optimal LV lead].							
Khatib et al. (26)	CRT	608	All-cause mortality	36	EAARN score	5 parameters, risk score of 0–5 points	One predictor, HR 3.28 (95% CI 1.37–7.8, *p* = 0.008); two, HR 5.23 (95% CI 2.24–12.10, *p* < 0.001); three, HR 9.63 (95% CI 4.1–22.60, *p* < 0.001); and four or more, HR 14.38 (95% CI 5.8–35.65, *p* < 0.001).
EAARN score’s calculation: LVEF < 22%, AF, Age ≥ 70 years, GFR < 60 mL/min/1.73 m^2^, NYHA IV. One point was attributed to each predictor.							
Brunet-Bernard et al. (39)	CRT	162	ΔLVESV ≥ 15% reduction after 6-month	6	L2ANDS2 score	5 parameters, risk score of 0–7 points	A score > 5 had a high positive likelihood ratio [+ LR (5.64), whereas a score < 2 had a high negative likelihood ratio (–LR (0.19)].
L2ANDS2 score’s calculation: LBBB (2 points), age > 70 years (1 point), non-ischemic origin (1 point), LVEDD < 40 mm/m^2^ (1 point), and septal flash (2 points).							
Rickard et al. (40)	CRT	879	All-cause death ± HTX ± LVAD	6	Early demise score	4 parameters, risk score of 0–4 points	The specificity for ≥ 2 and ≥ 3 risk factors was 72.6 and 94.6%, respectively.
Early demise score’s calculation: non-LBBB, pre-CRT LVEDD ≥ 6.5 cm, serum creatinine ≥ 1.5 mg/dL, and lack of β-blocker. One point was attributed to each predictor.							
Paoletti Perini et al. (41)	CRT-D	559	All-cause death ± HF hospitalization	72	CHADS_2_ and CHA_2_DS_2_-VASc score	7 parameters, risk score 0–9 points	CHA_2_DS_2_-VASc score (for HF hospitalization *p* < 0.013; for the combined event, *p* < 0.007), while the CHADS_2_ score was not independently associated with either the endpoints.
Calculation: CHADS_2_ score: congestive heart failure (1 point), hypertension blood pressure ≥ 140/90 mm Hg (1 point), age ≥ 75 years (1 point), diabetes mellitus (1 point), prior stroke, TIA or thromboembolism (2 points); and CHA_2_DS_2_-VASc score: congestive heart failure (1 point), hypertension blood pressure ≥ 140/90 mm Hg (1 point), age ≥ 75 years (2 points), diabetes mellitus (1 point), prior stroke, TIA or thromboembolism (2 points), vascular disease (1 point), age 65–74 years (1 point), female sex (1 point).							
Nauffal et al. (28)	CRT-D	305	All-cause death ± HTX ± LVAD	60	HF-CRT score	5 parameters, a score-system was created and divided into: category 1 (score 0–1), category 2 (score 2–3), and category 3 (score 4–5)	Patients with scores 0–1, 2–3, and 4–5 had a 3-year cumulative event-free survival of 96.8, 79.7, and 35.2%, respectively (log-rank, *p* < 0.001).
HF-CRT score’s calculation: hsCRP ≥ 9.42 ng/L, NYHA III/IV, creatinine ≥ 1.2 mg/dL, red blood cell count ≤ 4.3 × 106/μL, and cardiac troponin T ≥ 28 ng/L. One point was attributed to each predictor.							
Gasparini et al. (27)	CRT	5,153	All-cause mortality	60	VALID-CRT score	9 parameters, five quintiles. I: -1.841 - 0.061, II: 0.062 - 0.558, III: 0.559 - 0.937, IV: 0.938 - 1.364, V: 1.365 - 3.157	At 5 years, total mortality was 10.3, 18.6, 27.6, 36.1, and 58.8%, from the first to the fifth quintile.
VALID-CRT score’s calculation: 0.028 × age 66 - 0.044 × LVEF25 + 0.646 × AF1 - 0.154 × AF2 - 0.656 × ICD + 0.405 × GENDER + 0.317 × CAD + 0.844 × NYHA34 + 0.167 × diabetes. Where: age66 = age-66 years; LVEF25 = LVEF-25; AF1 = 1 if AF without AVJA is present, 0 otherwise (meaning both sinus rhythm or AF + AVJA); AF2 = 1 if AF with AVJA is present, 0 otherwise (meaning both sinus rhythm or AF without AVJA); ICD, CAD, NYHA III–IV, diabetes = 1 if present, 0 otherwise; gender = 1 if male, 0 if female.							
Bani et al. (21)	CRT	172	ΔLVEF ≥ 10% increase ± ΔLVESV ≥ 15% reduction after 6-month	24	Simplified Selvester Score (SSc)	The Simplified-SSc is created utilizing an ECG analysis. Patients are divided into 4 groups according to the presence of 0, 1, 2 or ≥ 3 points	The response rate was 85, 60, 60, and 50% within the 4 groups. Simplified-SSc was inversely correlated with response to CRT (*p* = 0.048).
SSc’s calculation: Lead I: R/S ≤ 1.5 = 1 point; Lead aVL: Q ≥ 50 ms = 2 points, R/S ≤ 1.0 = 1 point; Lead II: Q ≥ 30 ms = 1 point; Lead aVF: R/S ≤ 0.5 = 1 point; Lead V1: R ≥ 20 ms = 1 point, Lead V2: notch in the initial 40 ms of the QRS = 1 point; Lead V2: S/S’ ≥ 1.5 = 1 point; Lead V5: any Q = 1 point; Lead V6: R/S ≤ 2.0 = 1 point.							
Kang et al. (19)	CRT	93	ΔLVESV ≥ 15% reduction after 6-month	24		3 parameters, including strain analysis, risk score of 0–4 points	The sensitivity and specificity for prediction of a positive response to CRT at a score > 2 were 0.823 and 0.850, respectively (AUC: 0.92295% CI 0.691–0.916, *p* < 0.001).
Calculation: tricuspid annular plane systolic excursion ≥ 14.8 mm (2 points), longitudinal strain (LS) ≤ –7.22% (1 point), and complete LBBB with wide QRS duration (1 point).							
Seo et al. (11)	CRT	171	ΔLVESV ≥ 15% reduction after 6-month.	36	START score	6 parameters, including strain analysis, risk score (0–17 points)	A probability > 0.5 corresponded to a START score ≥ 10, and a probability > 0.9 corresponded to a score of ≥ 14.
START score’s calculation: 1 point for LBBB or RV pacing; mitral regurgitation index ≤ 40% was 2 points; use of beta-blocker, BUN ≤ 30 mg/dL, and LV dimension at end-systole ≤ 50 mm were 3 points, and CS-SD (standard deviation of time from QRS onset to the first peak on the circumferential strain curves) ≥ 116 ms was 4 points.							
Barra et al. (42)	CRT	638	All-cause mortality	60	Goldenberg risk score	5 parameters, two groups: risk score of 0–2 and score of ≥ 3	No significant differences in mortality rates were seen in patients with scores ≥ 3 (57.9% with CRT-D vs. 56.9% with CRT-P, *p* = 0.8).
Goldenberg risk score’s calculation: NYHA > 2, atrial fibrillation, QRS duration > 120 ms, age > 70 years, and BUN > 26 mg/dL. One point was attributed to each predictor.							
Höke et al. (29)	CRT	1,053	All-cause mortality	60	CRT-SCORE	15 parameters, risk groups: L5 [-4.42 – –1.60], L10 [-1.60 – -1.31], L20 [-1.31 – -0.82], L40 [-0.82 – -0.16], M [-0.16 – 0.28], H40 [0.28 – 0.79], H20 [0.79 – 1.18], H10 [1.18 – 1.44], H5 [1.44 – 2.89]	Estimated mean survival rates of 98% at 1 year and 92% at 5 years were observed in the lowest 5% risk group; whereas the highest 5% risk group showed poor survival rates: 78% at 1 year and 22% at 5 years.
CRT SCORE’s calculation: (−0.169 x AVJA) + (0.037 x Age) + (0.367 x Male gender) + (0.221 x Ischemic etiology) + (0.048 x AF) + (0.516 x diabetes mellitus) – (0.173 x LBBB) + (0.394 x NYHA class III) + (0.826 x NYHA class IV) – (0.156 x QRS duration ≥ 150 ms) – (0.013 x GFR) – (0.084 x Hemoglobin level) – (0.026 x LVEF) + (0.259 x Mitral regurgitation ≥ 3) + (0.325 x Restrictive LV function).							
Nauffal et al. (43)	CRT-D	305	HF hospitalization and appropriate ICD therapy	60	PROSE-ICD score	5 parameters, two score-systems were created and divided into: category 1 (score 0–1), category 2 (score 2), and category 3 (score ≥ 3)	Five-year cumulative risk of appropriate therapy was 4, 14.6, and 47.2% for score categories 1, 2 and 3, respectively (*p* < 0.001). Five-year cumulative risk of HF hospitalization was 21.1, 40.3 and 69.8% for score categories 1, 2, and 3, respectively (*p* < 0.001).
PROSE-ICD score’s calculation: predictors of appropriate ICD therapy: BUN > 20 mg/dL, hsCRP > 9.42 mg/L, no beta blocker therapy, and hematocrit ≥ 38%; predictors of HF hospitalization: atrial fibrillation, NYHA class III/IV, LVEF ≤ 20%, HS-IL6 > 4.03 pg/ml, hemoglobin < 12 g/dL. One point was attributed to each predictor.							
Wilkoff et al. (25)	ICD, CRT-D	57893 ICD and 67929 CRT-D.	All-cause mortality	36	Heart Rate (Hr) Score	Hr Score is determined from the atrial paced and sensed histogram	Hr Score 30–70% compared to Hr Score > 70% was associated with increased survival (CRT-D HR = 0.85; *p* < 0.001 and ICD HR = 0.88; *p* < 0.001).
Hr Score’s calculation: the height in the percentage of all beats in the tallest 10 beats/min rate histogram bin was defined as the Hr Score. Thus, if all beats were in one bin the Hr Score would be 100%.							
Nevzorov et al. (44)	ICD, CRT-D	2,617	All-cause mortality	12	AAACC score	4 parameters, risk score (0–10 points)	Mortality risk increased (from 1% with 0 point to 12.5% with > 4 points).
AAACC score’s calculation: age greater than 75 years (3 points), anemia (2 points), AF (1 point), chronic renal disease GFR < 30 min/mL/1.73 m^2^ (3 points) and chronic lung disease (1 point).							
Biton et al. (45)	ICD, CRT-D	756	All-cause mortality	12	MADIT-CRT score in mild HF	4 parameters, risk score (0–4 points)	1 point increase in the score was associated with two-fold increased mortality within the CRT-D arm (*p* < 0.001).
MADIT-CRT score in mild HF’s calculation: age ≥ 65, creatinine ≥ 1.4 mg/dL, history of CABG, LVEF < 26%. One point was attributed to each predictor.							
Providencia et al. (31)	CRT	1,301	ΔNYHA ≥ 1 improvement ± ΔLVEF ≥ 5% increase after 12-month	12	ScREEN score	5 parameters, risk score (0–5 points)	46.7% of patients with a score of 0 met the criteria for response, while 93.9% of individuals with a score of 5 were responders, *p* < 0.001.
ScREEN score’s calculation: female gender, GFR ≥ 60 mL/min/1.73 m^2^, QRS width ≥ 150 ms, LVEF ≥ 25%, NYHA ≤ 3. Each was assigned 1 point.							
Bakos et al. (46)	CRT	202	All-cause death ± HTX ± LVAD ± HF hospitalization.	36	CRT response score	Three 6-month response criteria formed a risk score	1 point increase was associated with a 31% decreased risk for the primary endpoint [HR 0.69 (95% CI: 0.50–0.96), *p* = 0.03].
CRT response score’s calculation: one point each for positive clinical (≥ 1 NYHA class improvement), echocardiographic (≥ 15% LVESV reduction) and biomarker (≥ 25% reduction in NT-proBNP) response 6 months after implantation.							
Végh et al. (22)	CRT	491	All-cause death ± HTX ± LVAD ± HF hospitalization	36	ECG score	Three post-implant ECG parameters were measured and compared to pre-implantation measurements, score (0–3)	The total score was an independent predictor for event-free survival [HR 0.65 (0.54–0.77) *p* < 0.001].
The predetermined ECG score was based on the standard 12-lead ECG, and included three parameters: (1) One point was assigned for a reduction of QRS width of at least 20 ms compared from baseline ECG to post-implant ECG. (2) One point was assigned for a reduction of at least 50% in the summed amplitude of R + S in lead V1 from baseline ECG to postimplant ECG. (3) One point was assigned if the intrinsicoid deflection point was identified within the first 40 ms from QRS onset at the follow-up ECG in the V1 lead.							
Maass et al. (24)	CRT	240	LVESVi reduction after 6-month	12	CAVIAR score	4 parameters (including vectorcardiography), risk score (0–9 points)	The predicted change of LVESVi: - 2 point = −1.3%, −1 point = −7.1%, 0 point = −12.5%, 1 point = −17.6%, 2 points = −22.4%, 3 points = −26.9%, 4 points = −31.2%, 5 points = −35.2%, 6 points = −38.9%, 7 points = −42.5%, 8 points = −45.8%, 9 points = −49.0%.
The CAVIAR score is the sum of the applicable values with minimum −2 and maximum 9 points. Age: year < 60 = 1 point, 60–74 years = 0 point, ≥ 75 years = −1 point; Vectorcardiographic QRS AREA: < 80 μVs = −2 points, 80–99 μVs = −1 point, 100–119 μVs = 0 point, 120–139 μVs = 1 point, 140–159 μVs = 2 points, 160–179 μVs = 2 points, 180–199 μVs = 3 points, 200–219 μVs = 4 points, ≥ 220 μVs = 5 points; Inter-ventricular mechanical delay < 15 ms = −1 point, 15–44 ms = 0 point, 45–74 ms = 1 point, ≥ 75 ms = 2 points; Apical Rocking: Absent = 0 point, Present = 2 points.							
Kisiel et al. (30)	CRT	552	All-cause mortality	108	AL-FINE score	6 parameters, risk score (0–6 points)	Overall mortality (C-statistics of 0.701) at seven years was in the range of 28% (0–1 points) to 74% (3–6 points).
AL-FINE score’s calculation: Age > 75 years, non-LBBB, Furosemide dose > 80 mg, Ischemic etiology, NYHA > III, LVEF < 20%. One point was attributed to each predictor							
Theuns et al. (47)	CRT-D	1,282	All-cause mortality	36	Risk Score	7 parameters, five quintiles: I: ≤ 0.3230, II: 0.3231–0.9044, III: 0.9045–1.4384, IV: 1.4385–2.0510, V: > 2.0510	Mortality ranged from 2.8% (lowest quintile) to 31.9% (highest quintile).
Risk Score’s calculation:0.656 × (MI) + 0.323 × (LVEF) + 0.641 × (COPD) + 0.992 × (CKD) + 0.941 × (hyponatremia) + 0.427 × (anemia) – 0.660 × (QRS150), where: LVEF = per 5% decrease of LVEF in patients with LVEF ≤ 35%. In patients with LVEF > 35%, the score associated with LVEF is 0; CKD = estimated GFR < 60 mL/min/1.73 m^2^, 1 if present, otherwise 0; Hyponatremia = serum level of sodium < 136 mmol/L, 1 if present, otherwise 0; Anemia = serum level of hemoglobin < 12 g/dL, 1 if present, otherwise 0; QRS150 = QRS duration ≥ 150 ms, 1 if present, otherwise 0; MI, COPD = 1 if present, otherwise 0.							
Feeny et al. (34)	CRT	925	ΔLVEF ≥ abs. 10% increase at 24-month	24		9 parameters, machine learning http://riskcalc.org:3838/CRTResponseScore/	Machine learning vs. guideline prediction AUC (0.70 versus 0.65; *p* = 0.012) and greater discrimination of event-free survival (concordance index, 0.61 versus 0.56; *p* < 0.001).
Calculation: QRS morphology (LBBB/RBBB/IVCD/RV-paced, QRS duration (ms), NYHA (1–4), LVEF (%) and end-diastolic diameter (mm), sex (male/female), ischemic cardiomyopathy (yes/no), atrial fibrillation (yes/no), and epicardial left ventricular lead (yes/no).							
Weber et al. (48)	CRT-D	720	Appropriate ICD therapy or death without prior appropriate ICD therapy (so-called prior death).	120		11 parameters, two risk scores. Risk cut-off values for prior death: low < 7, intermediate 7–10, high > 10; for appropriate ICD therapy: low < 0, intermediate 0–6, high > 6	Stratification according to predicted benefit translated into significantly different overall survival (*p* < 0.001) and correspondingly ranked survival curves.
Calculation: appropriate ICD therapy: NYHA functional class III/IV = 5 points, age at implantation = (–0.1 x Age) points, ischemic cardiomyopathy = 2 points, diuretic use = 5 points; Prior death: age at implantation = (0.1 x Age) points, male gender = 2 points, BMI ≥ 30 = 2 points, systolic blood pressure ≤ 100 mmHg = 2 points, impaired renal function (GFR ≤ 60 mL/min/1.73 m^2^) = 2 points, history of cancer = 3 points, peripheral artery disease = 3 points.							
Spinale et al. (10)	CRT	758	ΔLVESV ≥ 15 mL reduction after 6-month	12	Biomarker CRT Score	4 biomarkers, risk score (0–4 points)	Absolute change in LVESV (P < 0.001). 0 point: −30 ± 39, 1 point: −25 ± 50, 2 points: + 14 ± 43, 3 points: −13 ± 41, 4 points: −5 ± 36 mL.
Biomarker CRT Score’s calculation: sTNFr-II ≥ 7,090 pg/mL, sST-2 ≥ 23,721 pg/mL, hsCRP ≥ 7,381 ng/mL, and MMP-2 ≥ 982,000 pg/mL. One point value was assigned for each biomarker that exceeded the specific threshold.							
Manlucu et al. (33)	CRT-D, ICD	1,798	All-cause mortality	6	MAGGIC score	13 parameters, three risk categories: low:0–16 points, intermediate: 17–24 points, high: > 24 points. http://www.heartfailurerisk.org/	When patients were divided into 3 cohorts based on low, intermediate, and high MAGGIC scores, patients with high MAGGIC scores had lower 3-year survival rates than those with intermediate or low scores (73.0% versus 88.1% versus 96.8%; P < 0.001).
MAGGIC score’s calculation: input the following parameters to the online calculator: age (years), gender, diabetes, COPD, heart failure diagnosed within the last 18 months, current smoker, NYHA class, receives beta blockers, receives ACEi/ARB, BMI (kg/m2), systolic blood pressure (mmHg), creatine (umol/L), LVEF (%).							
Liu et al. (23)	CRT	387	ΔLVEF ≥ abs. 15% increase at 6-month	12	QQ-LAE Score	5 parameters, three risk categories	The proportion of super-response after 6-month CRT implantation in patients with scores 0–3, 4, and 5 was 14.6, 40.3, and 64.1%, respectively (*p* < 0.001).
QQ-LAE Score’s calculation: prior no fragmented QRS, QRS duration ≥ 170 ms, LBBB, left atrial diameter < 45 mm, and left ventricular end-diastolic dimension < 75 mm. One point was attributed to each predictor, and three score categories were identified.							
Cai et al. (49)	CRT and Afib	152	All-cause mortality and HF readmissions	60	Prognostic nomogram	5 parameters, nomogram https://pubmed.ncbi.nlm.nih.gov/32404049/#&gid=article-figures&pid=fig-3-uid-2	The C-index was 0.70 with a 95% CI of 0.61–0.78.
Prognostic nomogram’s calculation: NT-proBNP > 1,745 pg/mL, history of syncope, previous pulmonary hypertension, moderate or severe tricuspid regurgitation, thyroid-stimulating hormone > 4 mIU/L. Cross the line on the nomogram.							
Tokodi et al. (35)	CRT	1,510	All-cause mortality	60	SEMMELWEIS-CRT score	33 parameters, machine learning, online calculator https://arguscognitive.com/crt	AUC of the 5-year mortality was 0.803 (95% CI: 0.733–0.872, *p* < 0.001).
SEMMELWEIS-CRT score’s calculation: age at CRT implantation, gender, height, weight, medical history of hypertension, diabetes mellitus, type of atrial fibrillation (paroxysmal, persistent, permanent), NYHA, systolic blood pressure, LVEF assessed with two-dimensional echocardiography, etiology of heart failure (ischemic or non-ischemic), QRS morphology and width, type of the implanted device (CRT-P or CRT-D), current medical treatment with furosemide, other loop diuretics, thiazide diuretics, mineralocorticoid receptor antagonists, angiotensin-converting enzyme inhibitors and angiotensin II receptor blockers, beta-blockers, statins, amiodarone, allopurinol, digitalis, percentage of lymphocytes, glomerular filtration rate, hemoglobin concentration, serum levels of sodium, cholesterol, creatinine, urea and NT-proBNP.							
Patel et al. (50)	CRT	877	All-cause mortality	120		8 parameters, three risk categories (number of predictors > 1, > 3, > 5)	The sensitivity of factors > 5 was 0.65 with a specificity of 0.77 and a positive likelihood for survival of longer than 10 years of 2.83.
Calculation: Age < 65.53 years, LVEDD < 6.75 cm, QRS > 149 ms, BNP < 255 pg/mL, creatinine < 1.05 mg/dL, female sex, non-ischemic cardiomyopathy, no presence of atrial fibrillation. One point was attributed to each predictor.							
Yang et al. (51)	CRT in NICM	422	All-cause mortality or HTX	24	Alpha-score	5 parameters, three risk categories: (0–1 point = low, 2–3 points = intermediate, 4–5 points = high)	The cumulative survival free of the primary endpoint were 80%, 60%, 20% in the low, high, and intermediate-risk groups.
Alpha-score’s calculation: left atrial diameter > 44.5 cm, non-LBBB, NT-proBNP > 13.53 per 100 pg/ml, hsCRP > 2.87 umol/L, NYHA class IV. One point was attributed to each predictor.							
Milner et al. (52)	CRT or CRT upgrade	283	All-cause mortality	12	Modified Frailty Index (mFI)	11 parameters, frail if mFI ≥ 3	Frailty was associated with an increased risk of 1-year mortality (hazard ratio 5.87, *p* = 0.033).
Modified Frailty Index included non-activities of daily living independent, diabetes, COPD or congestive heart failure in the last 30 days, myocardial infarction within 6 months, previous percutaneous coronary intervention/CABG)/angina, hypertension, peripheral vascular disease, impaired sensorium, and TIA/cerebrovascular accident with or without deficits. The total number of components satisfied by each patient was added together to yield an integer score of 0 to 11.							
Liang et al. (36)	CRT	725	ΔLVEF ≥ abs. 10% increase at 1-year	12		19 parameters, machine learning, online calculator http://www.crt-response.com/	Ridge regression AUC = 0.77 (0.69–0.84); Support vector machine AUC = 0.76 (0.68–0.83); Logistic regression AUC = 0.77 (0.69–0.84).
Calculation: weight (kg), GFR (ml/min/1.73 m^2^), creatine kinase-MB (U/L), QRS duration (ms), left atrial diameter (mm), history of percutaneous coronary intervention (yes/no), amiodarone (yes/no), albumin (g/L), serum uric acid (mmol/L), free triiodothyronine (pmol/L), RR interval (ms), LVESD (mm), history of CABG (yes/no), aspartate transaminase (U/L), total cholesterol (mmol/L), free thyroxine (pmol/L), corrected QT interval (ms), LVEF (%), QRS morphology (LBBB/RBBB/IVCD/paced).							
Theuns et al. (53)	CRT-D	648	All-cause mortality	60	Heart Failure Meta-score	15 parameters, five quintiles. I: 0.64–1.75, II: 1.75–2.16, III: 2.16–2.59, IV: 2.59–3.05, V: 3.05–6.17, online calculator http://www.hfmetascore.org/HeartScore.aspx	Mortality ranged from 12% (95% CI, 7–20%) to 53% (95% CI, 44–62%), for quintiles 1 to 5, (overall log-rank *p* < 0.001).
Heart Failure Meta-score’s calculation: age (years), LVEF (%), creatinine (mg/dL), NYHA (1–4); male gender, African-American race, diabetes, COPD, peripheral vascular disease, ischemic cardiomyopathy, HF admission within 1 year before CRT, atrial fibrillation, wide QRS (≥ 120 ms), secondary prevention indication, history of ICD shocks.							
Younis et al. (12)	ICD, CRT-D	4,503	VT/VF and non-arrhythmic mortality	36	MADIT-ICD benefit score	12 parameters, three benefit groups. highest (score 76–100), intermediate (score 26–75), lowest (score < 25), online calculator https://redcap.urmc.rochester.edu/redcap/surveys/index.php?s=3H888TJ8N7	In the highest benefit group, the 3-year predicted risk of VT/VF was three-fold higher than the risk of non-arrhythmic mortality (20% vs. 7%, *p* < 0.001).
MADIT-ICD benefit score’s calculation: VT/VF (male, age < 75 years, prior non-sustained VT, HR > 75 bpm, systolic blood pressure < 140 mmHg, LVEF ≤ 25%, myocardial infarction, and atrial arrhythmia) and non-arrhythmic mortality (age > 75 years, diabetes mellitus, BMI < 23 kg/m^2^, LVEF < 25%, NYHA > II, ICD vs. CRT-D, and atrial arrhythmia).							
Zoni-Berisso et al. (54)	ICD, CRT-D	983	All-cause mortality	24	DECODE survival score index (SUSCI)	7 parameters, five risk groups according to the SUSCI (< 1, 1–4, 4–7, 7–10, and > 10)	The risk of death increased according to the severity of the risk profile ranging from 0% (low risk) to 47% (high risk).
DECODE SUSCI’s calculation: [(1.9359*ICM) + (2.2583* AGE ≥ 75) + (2.0295*INS) + (2.2369*NYHA) + (2.293*HOSP) + (1.7199*AF) + (2.1744*BMI)]. ICM [ischemic cardiomyopathy (0 = No; 1 = Yes)]; AGE [age at the time of device replacement/upgrade ≥ 75 years (0 = No; 1 = Yes)]; INS [insulin-dependent diabetes (0 = No; 1 = Yes)]; NYHA [0 = ≤ 2; 1 ≥ 3]; HOSP [hospitalization in the 30 days before the procedure (0 = No; 1 = Yes)]; AF [history of atrial fibrillation (0 = No; 1 = Yes)], and BMI < 26 kg/m2 [0 = No; 1 = Yes].							
Orszulak et al. (20)	CRT	49	ΔLVESV ≥ 15% reduction after follow-up	15	Regional Strain Pattern Index (RSPI)	Strain analysis, RSPI was calculated as the sum of dyssynchronous components	RSPI ≥ 7 points was a predictor of favorable CRT effect (OR: 12; 95% CI = 1.33–108.17; *p* = 0.004).
RSPI was calculated from all three apical views across 12 segments as the sum of dyssynchronous components. From every apical view, the presence of four components was assessed: (1) contraction of the early-activated wall; (2) prestretching of the late activated wall; (3) contraction of the early-activated wall in the first 70% of the systolic ejection phase; (4) peak contraction of the late-activated wall after aortic valve closure. Each component scored 1 point, thus the maximum was 12 points.							
Yamada et al. (55)	CRT	180	HF death amd lethal arrhythmic event	50	ALBI	2 parameters, ALBI score before CRT was High (> -2.60) or Low (≤ -2.60). The patients were then reclassified based on the ALBI score before and 6 months after CRT; High/High, High/Low, Low/High, and Low/Low ALBI groups.	High/High ALBI scores were an independent predictor of HF deaths compared with Low/Low ALBI scores (hazard ratio, 3.449, *p* = 0.008).
The ALBI score was calculated as follows: [log10 total bilirubin (mmol/L) × 0.66) + [albumin (g/L) × -0.085].							
Ikeya et al. (56)	CRT	263	All-cause mortality	31	CONUT	3 parameters, three groups according to the CONUT (0–1, 2–4, 5–12)	CONUT score ≥ 5 was significantly associated with all-cause mortality after adjusting for previously reported clinically relevant factors and the conventional risk score (VALID-CRT risk score) (all *p* < 0.05).
The CONUT score is the sum of the followings: serum albumin g/dL: 3.5–4.5 = 0 point, 3.0–3.49 = 2 points, 2.5–2.9 = 4 points, < 2.5 = 6 points; total lymphocytes/mL: > 1,600 = 0 point, 1,200–1,599 = 1 point, 800–1,199 = 2 points, < 800 = 3 points; cholesterol mg/dL: > 180 = 0 point, 140–180 = 1 point, 100–139 = 2 points, < 100 = 3 points.							
Saito et al. (57)	CRT	283	All-cause mortality	30	MELD-XI	2 parameters, three risk groups first tertile (MELD-XI = 9.44), second tertile (9.44 < MELD-XI < 13.4), and third tertile (MELD-XI ≥ 13.4)	The MELD-XI score was independently associated with mortality (adjusted hazard ratio: 1.04, 95% confidence interval: 1.00–1.07, *P* = 0.014).
MELD-XI score can be calculated as follows: 11.76 × ln (creatinine [mg/dL]) + 5.11 × ln (total bilirubin [mg/dL]) + 9.44.11. If a patient had a creatinine or total bilirubin level lower than 1.0 mg/dL, a value of 1.0 mg/dL was used to prevent negative logarithmic values in the formula.							
Maille et al. (32)	CRT-D	23 029	All-cause mortality	12	CRT-D Futility score	14 parameters, four risk groups: low (0–3), medium low (4–7), medium high (8–11), high (> 12).	The one-year mortality risk in the four groups were 1.7, 3.9, 8.1, and 16.6%.
The CRT-D Futility score can be calculated as: age (> 61 = 1 point, > 69 = 2 point > 75 = 3 point), undernutrition = 2 points, CKD = 2 points, liver disease = 2 points, anemia = 2 points, diabetes mellitus = 2 points, AF = 2 points, LBBB = minus 1 point, mitral regurgitation = 2 points, aortic stenosis = 2 points, history of hospital stay with heart failure = 2 points, history of pulmonary edema = 2 points.							

Δ6-min, changes in the 6-min walking test; Δdp/dt, measure of contractility; ΔLVEF, changes in the left ventricular ejection fraction; LVESV, changes in the left ventricular end-systolic volume; ΔNYHA, changes in the New York Heart Association functional class; ACEI, angiotensin-converting-enzyme inhibitor; AF, atrial fibrillation; ARB, angiotensin receptor blocker; AUC, area under the curve; AVJA, atrio-ventricular junctional ablation; BMI, body mass index; BNP, brain natriuretic peptide; BUN, blood urea nitrogen; CABG, coronary artery bypass graft surgery; CAD, coronary artery disease; CI, confidence interval; CKD, chronic kidney disease; COPD, chronic obstructive pulmonary disease; CRT, cardiac resynchronization therapy; CRT-D, cardiac resynchronization therapy with defibrillator; CRT-P, cardiac resynchronization therapy with pacing only; ECG, electrocardiography; GFR, glomelural filtration rate; HF, heart failure; HR, hazard ratio; hsCRP, high-sensitivity C-reactive protein; HS-IL6, high-sensitivity interleukin 6; HTX, heart transplantation; ICD, implantable cardioverter defibrillator; IVCD, intraventricular conduction delay; IVMD, interventricular mechanical dyssynchrony; LA, left atrium; LBBB, left bundle branch block; LV, left ventricle; LVAD, left ventricular assist device; LVEDD, left ventricular end-diastolic diameter; LVEDV, left ventricular end-diastolic volume; LVEF, left ventricular ejection fraction; LVESV, left ventricular end-systolic volume; LVESVi, indexed left ventricular end-systolic volume; LVGLS, left ventricular global longitudinal strain; MI, myocardial infarction; MMP-2, matrix metalloproteinase-2; NT-proBNP, N-terminal prohormone of brain natriuretic peptide; Num. of pat., number of patients; NYHA, New York Heart Association functional classification; OR, odds ratio; Publ. year, publication year; QRS, width of the QRS complex; RBBB, right bundle branch block; Ref, reference; RSD, radial strain delay; RV, right ventricular; RVFAC, right ventricular fractional area change; sST-2, soluble ST2 interleukin; sTNFr-II, soluble tumor necrosis factor receptor type II; TIA, transient ischaemic attack; VT/VF, ventricular tachycardia; ventricular fibrillation; x^2^, chi square.

The primary endpoint of the models was all-cause death or a composite of death in the majority of the cases (*n* = 33, 69%), otherwise, it was echocardiographic or clinical response to CRT (*n* = 15, 32%). The most commonly used variables in the models were ischemic etiology (*n* = 21, 44%), renal function (*n* = 21, 44%), age (*n* = 20, 42%), New York Heart Association classification (*n* = 18, 38%), LVEF (*n* = 15, 33%), QRS morphology (*n* = 15, 31%), QRS width (*n* = 14, 30%), atrial fibrillation (*n* = 13, 27%), gender (*n* = 13, 27%), and left ventricular dimensions (*n* = 12, 25%).

## Discussion

The very first risk score in CRT was developed by Heist et al. (6). It investigated the immediate hemodynamic response (improved contractility as assessed by the dP/dt of the mitral regurgitation jet) to CRT by using echocardiographic and electrophysiologic parameters (6). Following that, the Charlson comorbidity index (CCI) from Charlson et al. (7), was tested in 463 heart failure patients with CRT; a CCI score ≥5, meaning several comorbidities and worse overall state, reflected a more than 3 times mortality risk (8). In parallel, the MADIT-CRT score was created by Goldenberg et al. (9) by using the data of the 1,761 patients enrolled in the Multicenter Automatic Defibrillator Implantation Trial With Cardiac Resynchronization Therapy (MADIT-CRT). The MADIT-CRT identified the most relevant routine clinical risk factors that affect mortality in CRT: gender, etiology of heart failure, the presence of left bundle-branch block and wide QRS, prior heart failure hospitalizations, and left ventricular and atrial dimensions. The MADIT-CRT score has been served as a gold standard and used as a reference in many validation studies (10–12).

The Seattle Heart Failure Model (SHFM) is a well-known risk estimation tool to predict the 1-, 2-, and 5-year mortality in chronic heart failure patients with conservative therapy (13). Perrotta et al. (14) applied the SHFM to patients who received a CRT, or a CRT-D and the model showed a good discrimination capacity in the mortality prediction. In the same year, the SHFM was validated in CRT populations by others as well (15, 16). Park et al. (17) were the first who developed a risk score, the EchoCG score, by using echocardiographic strain analysis to predict the reverse remodeling after CRT implantation. Strain analysis was included in many models later (11, 18–20). Similarly, to strain analysis, electrophysiologic modalities were also used in risk score development, such as sophisticated ECG analysis (21–23), vectorcardiography (24), or heart rate histogram analysis (25).

However, simplicity and availability are the keys to risk score development. The EAARN (26), the VALID-CRT (27), the HF-CRT (28), the CRT-SCORE (29), the AL-FINE (30), the ScREEN (31), the CRT-D Futility score (32), the MAGGIC (33), and many others can be calculated with routine laboratory and clinical parameters. Incorporating these principal concepts, machine learning algorithms can provide personalized risk predictions and online calculators are also available (34–36).

## Conclusion

This is the first systematic review of risk scores in cardiac resynchronization therapy. The scores show a great diversity in terms of used predictors and endpoints. As we demonstrated, the number of the different scoring systems has drastically increased in the past few years and a very marked heterogeneity can be observed among them. Unfortunately, this makes their translation and transition into everyday clinical practice difficult. Furthermore, the majority of studies were conducted prior to the current era of quadruple HFrEF therapy. These limitations must be considered before the routine application of the score systems.

Rickard et al. have shown in a prior review that classic markers (native LBBB, non-ischemic etiology, wide QRS, female gender and sinus rhythm) predict outcomes after CRT-D (4). However, there is growing evidence available on novel risk factors for CRT response, incorporated into the numerous risk score systems. The predictors can be categorized into the following different groups: co-morbidities, clinical state, echocardiographic, electrocardiographic, routine blood markers, and novel biomarkers as shown in the present review; the overlap of the markers in the various models is minimal. Some biomarkers are not yet incorporated into the daily routine clinical practice and their widespread use is therefore limited. Moreover, the lack of cross-validation across the risk scores limits the ability to objectively determine which of them should be incorporated into daily practice.

Although all the listed risk scores have the potential to predict outcomes after CRT, more data is required to enable us to select which will be appropriate to use in the daily clinical practice to predict the prognosis of severe heart failure patients, who undergo CRT. As the number of possible predictors and combinations is overwhelming, machine learning based algorithms or the help of artificial intelligence might be required to develop a uniform CRT risk score system.

It must be emphasized that, currently, the decision of CRT implantation is based on the ejection fraction, the width of the QRS, and the presence of LBBB; none of the guidelines do endorse any risk score to be applied in the process. Therefore, risk scores are useful to give information regarding the prognosis after implantation but should not influence the implantation itself.

## Author contributions

AB and GS contributed to the conception and design of the study and wrote the first draft of the manuscript. GS and BM provided the institutional background to the study. AB and PP collected data and performed the statistical analysis. All authors contributed to manuscript revision, read, and approved the submitted version.

## Footnotes

1
https://www.ncbi.nlm.nih.gov/
